# The Burden of Disease Due to Road Traffic Noise in Hesse, Germany

**DOI:** 10.3390/ijerph18179337

**Published:** 2021-09-03

**Authors:** Janice Hegewald, Melanie Schubert, Matthias Lochmann, Andreas Seidler

**Affiliations:** 1 Institute and Policlinic of Occupational and Social Medicine, Faculty of Medicine, Technische Universität Dresden, 01307 Dresden, Germany; janice.hegewald@tu-dresden.de (J.H.); melanie.schubert@tu-dresden.de (M.S.); 2 Institute of Sociology, Faculty of Behavioral and Social Sciences, Chemnitz University of Technology, Thüringer Weg 9, 09126 Chemnitz, Germany; 3 Hessian Agency for Nature Conservation, Environment and Geology (HLNUG), Rheingaustraße 186, 65023 Wiesbaden, Germany; Matthias.Lochmann@hlnug.hessen.de

**Keywords:** environmental noise, burden of disease, road-traffic noise, cardiovascular disease, depressive disorders, noise annoyance, sleep disturbance, disability-adjusted life years DALYs, Germany

## Abstract

Road-traffic-noise exposition is widespread in Germany and can have harmful health effects. As guidance for informed decision-making, we estimated the environmental burden of disease attributable to road-traffic noise in Hesse, Germany as disability-adjusted life-years (DALYs). Using detailed road-traffic-noise exposure data provided by the Hessian Agency for Nature Conservation, Environment, and Geology (HLNUG), we calculated the DALYs due to road-traffic noise > 40 dB(A) L_24h_ (unweighted average 24 h noise level) and other noise metrics for endpoints with known dose-response functions and evidence in the literature (NORAH-study on disease risks and WHO reviews): cardiovascular disease, depressive disorders, road-traffic annoyance, and sleep disturbance. We calculated the population-attributable fractions (PAF) for road-noise-related cardiovascular disease (hypertensive heart disease, ischemic heart disease, and stroke) and depressive disorders in the population using published relative risk estimates. We multiplied the PAFs with the Hessian proportion of the 2015 WHO DALY estimates for Germany in people aged ≥ 40 years. For high annoyance and high sleep disturbance, we used published dose-response functions to determine the burden for residents of all ages. For Hesse, we found a total of 26,501 DALYs attributable to road-traffic noise or 435 DALY per 100,000 persons for the reference year, 2015. Further, we estimated that a hypothetic uniform road-traffic-noise reduction of 3 dB would prevent 23% of this burden of disease.

## 1. Introduction

Road-traffic noise is the largest source of noise pollution in Europe by far [1]. According to the World Health Organization (WHO), about 40% of the European population is exposed to road traffic-weighted day–evening night-noise levels (L_DEN_) of ≥55 dB(A) [2]. Exposure to road-traffic noise has short- and long-term adverse effects on physical and mental health and well-being. Cardiovascular and metabolic effects [3], sleep disorders [4] as well as mental disorders [5], and severe annoyance [6] are associated with prolonged exposure to road-traffic noise.

One method of quantifying health loss is to calculate disability-adjusted life years (DALYs). DALYs are a measure that sums the projected years of life lost due to mortality and years of healthy life lost due to non-fatal morbidity. The WHO and the World Bank introduced this concept in their Global Burden of Disease (GBD) study to quantify and compare health loss in different world regions [7,8]. DALYs aid decision-making and help to derive recommendations for intervention measures.

Methods to calculate the DALYs attributable to specific risk factors also can be used to quantify the negative impact of environmental risk factors on human health, or the “Environmental Burden of Disease” [9,10,11]. The environmental burden due to air and water pollution, food contamination, injuries, and unsafe traffic, as well as traffic noise, is estimated to account for 23% (95% confidence interval (CI) 13–34%) of the total disease burden [12].

A decade ago, Babisch and Kim [13] determined the road-traffic-noise-related disease burden for myocardial infarction in Germany for the WHO reports “Burden of disease from environmental noise” [2] and “Environmental burden of disease associated with inadequate housing” [14]. In sum, myocardial infarction caused by road-traffic noise resulted in a total loss of 30,147 healthy life years (DALYs) for the reference year considered. Based on data from the Augsburg KORA (Cooperative Health Research in the Augsburg Region) cohort study, the VegAS study of the German Federal Environment Agency (Umweltbundesamt) calculated the traffic noise-related disease burden for annoyance, sleep disorders, heart attacks, strokes, and arterial hypertension [15]. This study observed the highest values for road-traffic noise-related hypertension with 45,570 DALYs (264.73 per 100,000) for metropolitan areas with more than 250,000 inhabitants. Strokes attributable to road-traffic noise resulted in 35,246 DALYs (204.75 per 100,000) and myocardial infarctions in 3118 DALYs (18.11 per 100,000). Additionally, 11,948 DALYs (69.41 per 100,000) were attributed to annoyance from road-traffic noise. There were 17,684 DALYs (102.74 per 100,000) for sleep disorders caused by road-traffic noise. In addition, the Environmental Burden of Disease in European countries (EboDE) study evaluated data on the environmental burden of disease in six European countries (Belgium, Finland, France, Germany, Italy, and The Netherlands) [10,16]. For Germany, there were almost 60 DALYs per 100,000 for sleep disorders caused by traffic noise.

More recently, Tobollik, et al. [11] used the 2017 EU Environmental Noise Directive (2002/49/EC) noise mapping of Germany to estimate the DALYs attributable to noise exposures of L_DEN_ ≥ 55 dB(A) and L_night_ ≥ 50 dB(A). Another recent study by Schreckenberg, et al. [17] estimated the DALYs for Düsseldorf, Germany (a city with around 620,000 residents) with L_DEN_ noise exposures ranging from 40 to 78 dB(A). This study estimated 1301 DALYs (242 per 100,000) due to coronary heart disease, annoyance, and sleep disturbances among adults in Düsseldorf were attributable to road-traffic noise exposure.

The aim of our study is to estimate the burden of disease (outcome) in 2015 (time) caused by road-traffic noise (exposure) for the general population of Hesse, Germany (population) and the potential impact of noise-abatement measures on health. With over 6 million residents, Hesse is the fifth most populous of the 16 German states. Hesse comprises both large urban areas like Frankfurt, Wiesbaden, Darmstadt, and Kassel, as well as expansive rural regions. According to the 2016 European Statistics on Income and Living Conditions (EU-SILC) survey, 29% of Hessian respondents complained that noise due to neighbors, traffic, and industry was a problem in their homes [18]. This percentage was higher than the German average of 25% and the European average of 18%. Road traffic noise is a substantial contributor to noise in many residential areas. In the 2012 “German Health Update” study (Gesundheit in Deutschland Aktuell, GEDA), participants most frequently cited road traffic as being a source of disturbing or annoying noise, followed by noise from neighbors [19].

We set out to estimate the DALYs attributable to road-traffic noise using, to our knowledge, the most accurate exposure data available for road-traffic noise L_DEN_ > 40 dB(A) at each residential building in Hesse. As the one of several sensitivity analysis scenarios, we compare our results with the DALYs calculated based on the EU Environmental Noise Directive (2002/49/EC) mapping of selected large cities and major roads. The EU’s noise mapping depicts only the proportion of the population exposed to L_DEN_ > 55 dB(A) and L_night_ > 50 dB(A) (or electively L_night_ > 45 dB(A)).

A further aim is to examine the potential health benefit of reducing road-traffic noise. Measures that encourage quieter (and healthier) modes of transportation, such as bicycling and walking, as well as lowering urban speed limits to 30 km/h (18.6 mph) and using “silent road surfaces” help reduce road-traffic noise. Also, the ongoing transition from gasoline and diesel motor vehicles to electric vehicles is associated with a noise reduction of 3 to 4 dB(A) in urban areas [20,21]. Thus, we also examine how a 3-dB(A) reduction of road-traffic noise in Hesse would impact the road-traffic-related burden of disease.

## 2. Materials and Methods

### 2.1. Noise Exposure Estimation

Road-traffic-noise exposure data came from the 2017 “PLUS-Mapping” of Hesse provided by the Hessian Agency for Nature Conservation, Environment, and Geology (HLNUG) [22]. In contrast to noise estimations based on the European Union (EU) Environmental Noise Directive (Directive 2002/49/EC), which calls for noise assessments on major roads with at least 8219-vehicle passages per day, the “PLUS-mapping” uses information from all roads with vehicle passage data to map noise levels as low as 40 dB(A). Although this is the most detailed and extensive Hessian street-noise mapping with the most realistic exposure data to our knowledge, it is still influenced, e.g., by missing data on the smallest streets and certain inaccuracies of the sound-propagation calculations. Therefore, the precise form of our exposure data, especially at the lower end of our exposure range, should be interpreted with some care.

The proportion of the population exposed to noise L_DEN_ ≥ 40 dB(A) was estimated using 2011 census data (accurate to 100 × 100 m^2^) updated with municipality-specific population information from DESTATIS from 2013. Residents of municipalities were distributed to appropriate house perimeters of the official real estate cadaster information system (Amtliches Liegenschaftskatasterinformationssystem) from January 2014 [23]. All house residents were assigned noise values for the most exposed (loudest) façade.

The noise data did not include information for two smaller rural communities: Frielendorf (population 7330) and Gilserberg (population 3083). We imputed the missing data using the exposure data from two similarly sized rural communities with a comparable age structure: Calden (population 7335) and Hohenroda (population 3078). Even if this imputation slightly overestimated the traffic noise exposure in Frielendorf, as Calden is located just south of the Kassel Airport, and may experience additional (terrestrial) airport traffic, the relatively small size of this community should make any effect on the overall PAFs and DALYs negligible.

Depending on the respective dose-response functions, we used either the unweighted average 24 h noise level L_24h_ or L_DEN_ to determine the PAFs and L_DEN_ to assess the prevalence of highly road-traffic annoyed residents. We estimated the prevalence of noise-related sleep disorders using nightly (22:00 to 6:00) average noise levels (L_night_).

### 2.2. Selection of Outcomes (Clinical Endpoints)

We selected the health outcomes for our DALY calculations based on systematic reviews conducted for the “WHO Environmental Noise Guidelines for the European Region” project [3,4,6,24,25,26,27,28,29,30] and on more recent evidence for depression [5,31].

We determined sufficient evidence for a relationship between road-traffic noise exposure and the following outcomes: annoyance, sleep disorders, ischemic heart disease, and stroke. We did not consider hypertension because van Kempen, et al. [3] concluded there is only “very low” evidence for a relationship between incident hypertension and road-traffic noise (relative risk [RR] = 0.97, 95% CI 0.90–1.05).

The WHO reviews on cognition [24] and mental health [25] found no clear evidence of an association with road-traffic noise. However, these reviews (which included studies only up to 2015) did not conduct a meta-analysis. The Noise-Related Annoyance, Cognition, and Health (NORAH) Study on Disease Risks [32] was published after the WHO review’s literature search was completed and indicates an association between traffic-noise exposure and depressive disorders. In this study, based on a Hessian study population, we found a clear dose-response relationship between road-traffic noise and depression with a 4% risk increase (95% CI 3–5%) per 10 dB increase of road-traffic noise. Also, the results of two more recent systematic reviews with meta-analyses [5,31] (including our own review on the influence of traffic noise on mental health [5]) find increased risks for depression associated with increasing road-traffic noise that are approaching statistical significance. Although Dzhambov and Lercher [31] graded the confidence of evidence as “very low”, the odds of depression were found to be statistically significant at noise above 55 dB(A). Our own review found four high-quality prospective studies published since 2016 that reported risks ranging between 1.04 and 1.28 [32,33,34,35], although only half of these results achieved statistical significance [5,32,33]. A newer prospective study from Switzerland also found an increased risk of depression (RR = 1.06, 95% CI 0.93–1.22) that did not achieve statistical significance (possibly in part due to the low incidence of depression in the cohort: 11 cases per 1000 person-years) [36]. Nevertheless, we decided to include depression in our core analyses to determine depression attributable to road-traffic noise.

### 2.3. Population Attributable Risk Fractions (PAF)

For our core analysis, we calculated the PAFs for all noise levels above 40 dB(A) L_24h_ using 40 dB(A) L_24h_ as the counterfactual value. PAFs for cardiovascular diseases and depressive disorders (clinical endpoints) were calculated using the following formula [37,38]:

(1) $$\mathrm{PAF}=\frac{\sum_{i=1}^{n}p_{i}\times (RR_{L24h,i}-1)}{1+\sum_{i=1}^{n}p_{i}\times (RR_{L24h,i}-1)}$$

Here, *i* stands for each noise level (0.1 dB-steps used), *n* is the total number of noise levels, *p_i_* is the proportion of the population exposed to a noise level, and *RR*_L24h,i_ is the relative risk for the specific noise level. According to Rockhill, et al. [39], this PAF formula might be inadequate when confounding exists. In the case of confounding, a formula based on the exposure distribution among cases and the adjusted relative risk will produce an internally valid PAF estimate. Although this alternative formula might be preferable for the confounded relationships between road-traffic noise and disease, we do not have information on the exposure distribution among cases in Hesse. Thus, we used the above formula as the best possible approximation.

We used the risk estimates adjusted for age, sex, education and occupation (when available: In the NORAH study on health risks, this information was only available for one-third of the insured population), and the regional proportion of the population receiving social assistance from the NORAH study on health risks to calculate PAFs. The NORAH study population included persons aged ≥ 40 years who were insured with one of the three large statutory health insurers in and around the Frankfurt am Main area (administrative district of Darmstadt, the cities of Mainz and Worms, and the rural districts of Mainz-Bingen and Alzey-Worms). The NORAH study population (*n* = 1,026,670) comprised about 23% of the over 40-year-olds in the study area [32,40,41,42,43,44,45]. Not only did the geographical area of the NORAH study region include a part of Hesse, but cultural and social similarities of the NORAH study population with the general Hessian population aged ≥ 40 years should make the risk estimates of the NORAH study on health risks representative for Hesse. Additionally, the NORAH study of health risks considered noise exposure from a starting point of 40 dB(A), which corresponds well to the exposure data available from the “PLUS-Mapping” of Hesse. Pooled analyses include studies with varying ranges of exposure levels. The relative risks for cardiovascular diseases (i.e., hypertensive heart disease and heart failure [ICD-10: I11, I13.0, I13.2, I50], myocardial infarction [ICD-10: I21], stroke [ICD-10: I61, I63, I64]) and depressive disorders (ICD-10: F32, F33, F34.1, F41.2) per 10 dB(A) are shown in Table 1. As a first sensitivity analyses (Sens1), we also considered the WHO Environmental Noise Guideline pooled risk estimate for incident ischemic heart disease (Sens1a) and stroke (Sens1b) [3] using a counterfactual of L_DEN_ = 53 dB (the weighted average of lowest noise levels included in the pooled analysis) [30]. Ischemic heart diseases and stroke comprised 49.6% and 19.0% of the WHO DALYs due to all cardiovascular diseases in Germany in 2015, respectively [46].

We also calculated the PAF corresponding with the upper and lower bounds of the published 95% confidence intervals describing the variance (parameter uncertainty) of the relative risk estimates (Table 1).

### 2.4. DALYs—Cardiovascular Disease and Depression

We used the total (all risk, cause specific) WHO burden of disease calculations for Germany for 2015 to estimate the DALYs for cardiovascular diseases and depressive disorders in Hesse [46]. We chose to use the 2015 DALYs because the noise-exposure estimates originate from the 2017 noise mapping of Hesse [47] and the 2013 population estimates.

DALYs are calculated by adding the years of life lost (YLL) from mortality to the years of life lived with a disability (YLD), attributable to a disease or disorder, during a given year.

(2) DALY = YLL + YLD

The WHO calculated YLL by multiplying the number of age-specific deaths (N) due to a specific cause within a given year with the corresponding years of life lost (L) based on a standard reference table of life-expectancies (using the highest projected life-expectancy at birth 90 years). No age weighting or discounting for time was applied [8].

(3) YLL = N × L

The WHO multiplied the disease prevalence (*p*) with disability weights (DW) to estimate the cause-specific loss of healthy years or YLD. DWs take on values ranging from 0 (worst possible health) to 1 (perfect health) and quantify the proportion of health or well-being lost due to a disorder [8]:

(4) YLD =p× DW

Basing our calculations of burden of disease attributable to road-traffic noise on the WHO values has the added benefit that the WHO correct YLD values for common comorbidities. This is done to prevent the double-counting of comorbid conditions, which can lead to a person with multiple conditions losing more than one year of disability-adjusted life years per year. The WHO methods report [8] states, “YLDs by cause at age, sex, country, and year levels were adjusted for comorbidity with simulation methods”.

The DALY estimates for 2015 [46] that served as the basis of our calculations are shown in Table 2. Cardiovascular diseases due to hypertensive heart disease (ICD-10: I10-I15), ischemic heart disease (I20-I25), and stroke (I60-I69) caused a total of 3,963,058 DALYs in Germany. Depressive disorders (major depressive disorder and dysthymia) caused 586,898 DALYs in Germany. The NORAH study estimated the disease risks in the population ≥ 40 years of age, so we applied our estimation to the DALYs estimation in the population ≥ 40 years of age. To obtain the DALYs for the German population aged over 40 years, we added the DALYs for the age categories 50–59 years, 60–69 years, and 70+ years with half of the DALYs in the 30–49 years category. We estimated the risk-independent, cause-specific DALYs for Hesse, assuming a uniform distribution of DALYs across Germany. Hesse comprises about 7.45% of the German population [48], which corresponds to 287,321 DALYs for cardiovascular disease (of which 187,138 are from ischemic heart disease) and 27,603 DALYs for depressive disorders in the population aged 40 years and over.

We determined the burden of disease attributable to road-traffic noise (DALY_road_) for the clinical endpoints by multiplying the total $DALY_{i}$ for ≥40 year olds in Hesse by the corresponding $PAF_{i}$ and adding them up as follows [49]:

(5) $$\mathrm{DALY}{\mathrm{road}}_{\;}=\sum_{i=1}^{n}PAF_{i}\cdot DALY_{i},$$

with i the index for the different clinical endpoints. We considered *n* = 2 clinical endpoints, i.e., cardiovascular diseases and depressive disorders.

### 2.5. DALYs—Highly Annoyed and Highly Sleep-Disturbed

The DALYs for annoyance and sleep disturbance due to road-traffic noise were calculated by estimating the percentage of highly annoyed (%HA) and highly sleep-disturbed (%HSD) persons. We used the formulas published in the most recent WHO Environmental Noise Guidelines for the European Region reviews to estimate %HA [6] and %HSD [4]. In the case of %HA, two functions are provided: either based on a meta-regression of all available studies or excluding study results from the Alps (due to unique acoustic characteristics in valleys) and from Asia (which examined a restricted range of noise) [6]. Both are quadratic functions starting with an increased level of annoyance at L_DEN_ = 40 dB(A) and declining to minima between 45 and 48 dB(A). We would have expected functions to start at a relatively low annoyance level near the counterfactual value and increase strictly monotonically. Thus, we chose the function which excluded the Asian and Alpine studies because this function is lower, i.e., more conservative in the range 40–55 dB(A), and the—in our opinion—problematic functional form had less impact on the calculation of burden of disease:

%HA = 116.4304 − 4.7342(L_DEN_) + 0.0497(L_DEN_^2^) (6)

The percentage of highly sleep-disturbed residents was estimated from the following formula [4], which is valid for L_night_ ranging from 40–65 dB:

%HSD = 19.4312 − 0.9336(L_night_) + 0.0126(L_night_^2^) (7)

We then estimated the number of road noise-annoyed residents (all ages) as follows:

(8) $$\mathrm{Total}\;\mathrm{highly}\;\mathrm{annoyed}=\overset{n}{\underset{i=1}{\sum }}p_{i}\cdot r_{i}$$

where $r_{i}$ is the number of Hessian residents exposed to a certain level of noise (i), and $p_{i}$ is the %HA expected for the *i*-level of noise. For annoyance we used the noise metrics L_DEN_ starting with $r_{1}$ at 40 dB(A) and using L_DEN_ < 40 dB(A) as counterfactual. We calculated the number of severely sleep-disturbed persons analogously with nightly noise levels (L_night_, 22–6 h) of 40.0 dB(A) and above using L_night_ < 40 dB(A) as the counterfactual. Persons under the thresholds of L_DEN_ < 40 or L_night_ < 40 dB(A) did not contribute to the proportion of people who were highly annoyed.

The YLD was the product of this prevalence and the DWs for annoyance and sleep disturbance, respectively. We used the DW of 0.02 for being highly annoyed and the DW of 0.07 for sleep disturbances [2,11]. Since annoyance and sleep disturbance do not result in mortality, the YLD is equivalent to the DALY. No confidence intervals were provided with the %HA and %HSD formulas, so we were unable to calculate confidence intervals (parameter uncertainty) for these estimates.

In a second sensitivity analysis (Sens2), %HA was calculated using the formula from Guski, et al. [6], based on a meta-regression of all available studies, including studies from the Alps and Asia:

%HA_sensitivity_ = 78.9270 − 3.1162(L_DEN_) + 0.0342(L_DEN_^2^) (9)

### 2.6. Comparison with EU Environmental Noise Directive Noise Estimates

**Figure 1:**
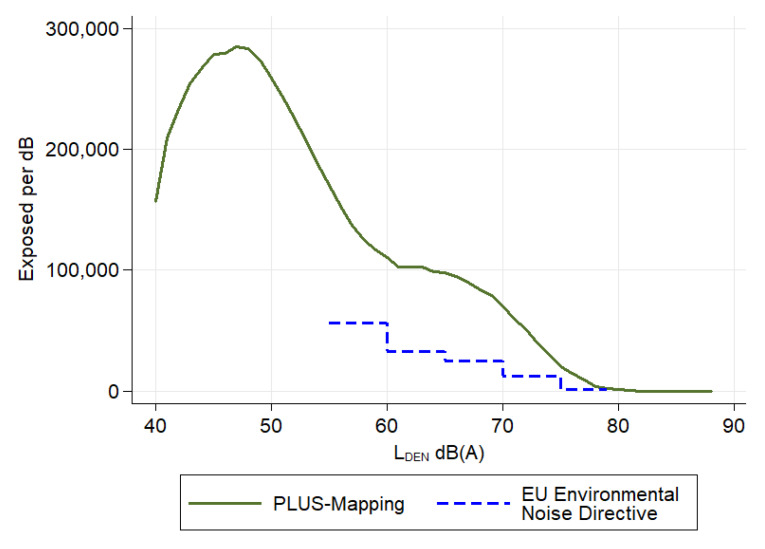
Number of road-traffic-noise-exposed residents (L_DEN_) in Hesse, Germany based on 2017 “PLUS-Mapping” [22] or the EU Environmental Noise Directive mapping of Hesse, Germany. According to the “PLUS-Mapping” 619,571 residents were not exposed to road noise (L_DEN_ ≤ 40 dB[A]). Using the EU Environmental Noise Directive mapping, 5,474,104 residents were not exposed to road noise (L_DEN_ < 55 dB[A]).

**Figure A1:**
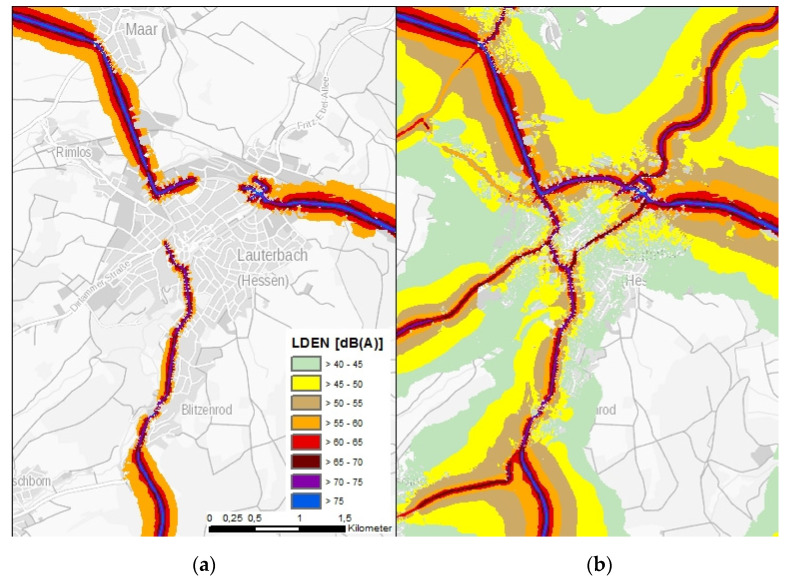
Maps of road-traffic noise exposure in L_DEN_ for one location (Lauterbach, Hesse) (**a**) according to the European guidelines and (**b**) according to the “PLUS-mapping” [67].

We also compared the DALYs estimated with the more accurate “PLUS-Mapping” of Hesse with those based on EU Environmental Noise Directive noise estimates in a further sensitivity analysis (Sens3). For these comparisons, we used data for the EU mapping published by the HLNUG, shown in Figure 1 with the exposure distribution, according to the “PLUS-Mapping” [22]. An example acoustic map, showing the difference between the EU mapping and the “PLUS-Mapping” of road-traffic noise, is shown in Appendix A, Figure A1. For the purposes of the comparison, we assumed that all of the remaining residents not included in the EU noise mapping of Hesse were exposed to noise under 55 dB(A). Using the total population reported in the “Final Report Environmental Noise Mapping Hesse 2017” (N = 6,116,203 as of 30 June 2015), 5,474,104 (90%) residents were not included in the EU noise mapping and could be assumed to have road-noise exposures below 55 dB(A) [47].

In order to be able to use the L_DEN_-exposure data with the dose-response functions derived for unweighted continuous sound pressure levels over 24 h ($L_{24h}$), we transformed the exposure data using the conversion, $L_{\mathrm{DEN}}-3{.3=L}_{24h}$ [50].

### 2.7. Impact of a 3 dB Noise Reduction

We considered the impact of future noise reductions by subtracting 3 dB(A) from the noise-exposure estimates (L_DEN_ and L_night_) in an additional analysis. Reduced L_DEN_ and L_night_ values under 40 dB(A) did not contribute to health risks. We used the reduced noise exposure estimates to recalculate the PAFs for cardiovascular diseases and depression and the prevalence of annoyance and sleep disturbance.

## 3. Results

### 3.1. Cardiovascular Disease

Using the “PLUS-Mapping” road noise exposure data for Hesse and an exposure-risk relationship for incident cardiovascular disease of 2.4% per 10 dB(A) resulted in a PAF of 2.1% (95%CI 1.4% to 2.8%) (Table 3 and Table 4). Thus, 5970 DALYs due to cardiovascular disease were attributable to road-traffic noise in Hesse. In their review, van Kempen, et al. [3] reported that the risk for incident ischemic heart disease increased by 8% per 10 dB(A). This larger increase may have been caused by a number of pooled studies considering risk increases to higher noise exposures, beginning around 50 dB(A). Using this exposure-risk relationship (Sens1a) but with the higher counterfactual of 53 dB(A), resulted in a PAF of 2.5% and 4739 DALYs (137.4 per 100,000). This also applies to stroke, which van Kempen, et al. [3] examined stroke separately. For stroke an even higher increase in stroke risk of 14% per 10 dB(A) was reported. The analogous calculations with stroke resulted in a PAF of 4.4% and 3151 DALYs (91.3 per 100,000).

### 3.2. Depressive Disorders

The proportion of depressive disorders attributable to road-traffic noise was 3.5% (95% CI 2.7% to 4.3%). This resulted in 971 road-traffic noise-related DALYs for Hesse in persons ≥40 years of age (Table 3 and Table 4).

### 3.3. Highly Annoyed

Based on exposure to road-traffic noise L_DEN_ ≥ 40 dB(A) in Hesse, 490,000 (8.04%) residents were highly annoyed by road-traffic noise. This loss of well-being (disability weight = 0.02) resulted in 9800 DALYs. Higher values resulted from the annoyance-curve based on aggregated data including Alpine and Asian studies (Table 5; Sens2).

### 3.4. Highly Sleep Disturbed

An estimated 138,427 people were highly sleep-disturbed and had either difficulty falling asleep or experienced awakenings due to road-traffic noise in Hesse. This reduced state of health due to sleep disturbances resulted in 9760 DALYs for Hesse’s noise-exposed residents (Table 6).

### 3.5. DALYs Based on EU Environmental Noise Directive Mapping

Using the road-traffic noise exposure distribution based on the EU Environmental Noise Directive mapping for our sensitivity analysis 3 (Sens3), we obtained lower PAFs of 0.5% for cardiovascular diseases and 0.8% for depressive disorders. This also reduced DALYs for cardiovascular diseases and depressive disorders by around 76% (Table 7).

DALYs for high annoyance decreased by 79% (in Sens3) when only noise exposures (>55 dB(A) and higher mapping thresholds) from the EU noise mapping were considered (Table 8). Using the EU values from Hesse for nightly noise exposure >45 dB(A) reduced the DALYs for sleep disturbances by 70%. However, the EU Noise Directive sets the threshold for nightly noise exposure at 50 dB(A). The reporting of L_night_ values below <50 dB(A) in Hesse was optional. Excluding DALYs for noise exposures under 50 dB(A) resulted in 1984 DALYs and a DALY reduction of 80%.

### 3.6. Total Burden and 3-dB Reduction Scenario

A global 3-dB reduction in noise (additional analysis) decreased the PAF for cardiovascular disease from 2.1% to 1.6%. This percentage change resulted in an absolute reduction in DALYs of 1426 for cardiovascular diseases among ≥40-year-olds in the reference year (Table 9). The noise reduction attenuated the PAF for depressive disorders from 3.5% to 2.7% and would prevent 236 DALYs. The most considerable reduction in DALYs resulted from sleep disturbances following a 3-dB reduction in L_night_. In total, assuming independency of the regarded outcomes, a 3-dB reduction in noise would reduce the DALYs by 25% (from 26,501 to 19,884). We stress that the form of the annoyance function (quadratic function with a minimum at about 48 dB) makes it poorly suited for this form of reduction calculation. However, as the relative change in burden coincidently results in a reduction similar to the other outcomes, we have included it anyhow.

## 4. Discussion

According to our calculations, cardiovascular disease, depressive disorders, annoyance, and disturbed sleep due to road-traffic noise cost the roughly 6 million residents of Hesse 26,501 years of healthy life (DALY) in 2015. This equates to about 4.3 years of healthy life lost per 1000 persons (population of Hesse in 2015 = 6,093,888 [51]). The burden of disease, calculated with the official EU Environmental Noise Directive-exposure data, assumes the population not included in this mapping is completely unexposed to noise. This underestimates around 76% of the burden of disease due to cardiovascular disease and depressive disorders attributable to noise. We also determined that an overall 3-dB(A) reduction in road-traffic noise would prevent nearly a fourth of the road-noise attributable burden of disease calculated using the “PLUS-Mapping” exposure data.

### 4.1. Comparison to Previous Burden of Health Studies

Although the DALY-concept facilitates comparisons of fatal and nonfatal burden of disease between populations, comparing DALYs attributable to environmental factors is challenging. Differences in the estimations and assumptions (e.g., disability weights, duration of health states, life-expectancy) used to calculate DALYs reduce the comparability of estimates. Also, numerous postulations are needed to determine noise-related health loss. The first of these assumptions is the selection of outcomes associated with noise. In this respect, our study differed from other studies in that we also estimated the proportion of depressive disorders attributable to road-traffic noise. Previous calculations of road-traffic related burden of disease did not consider depressive disorders [11,17]. However, the lack of consideration of road noise-related depression risks would only result in a 4% lower DALYs estimation (reducing the DALYs from 26,501 to 25,530). We also included hypertensive heart disease, heart failure (ICD–10: I10–I15) and stroke (I60–I69), in addition to ischemic heart diseases.

In contrast, some previous studies considered disease burden due to hypertension [15,52]. At the time of the VegAS study, road-traffic noise appeared to cause a sizable increase in hypertension [15]. More recent research indicates that the body of evidence for an increased risk of hypertension due to road-traffic noise is uncertain [3] and newer DALY calculations [17,53] omit hypertension. Moreover, arterial hypertension usually presents without symptoms, and although it is a critical risk factor for other cardiovascular diseases, the majority of milder cases will not contribute directly to the burden of disease [54].

Another difference between studies on burden of disease related to environmental noise is the noise exposure levels considered. Unlike some estimates of road-traffic-related burden of disease [15,52,53], we had information on the proportion of people exposed to road-traffic noise between 40 and 55 dB(A). There is evidence of increased disease risks at these lower noise levels [4,6,32,42,43,44,45]. Although the increase in risk at road-traffic noise between 40 and 55 dB(A) L_DEN_ is low, a large proportion of the population are exposed to noise between 40 and 55 dB(A) (Figure 1). This increased proportion of persons to low noise increases the proportion of disease attributable to road-traffic noise. Our DALY estimates may also not be compared with the calculations of Tobollik, et al. [11]. Tobollik, et al. [11], in de facto terms, assume that the noise-related DALYs per 100,000 inhabitants in noise-exposed (according to the EU Environment Directive L_DEN_ > 55 dB(A)) areas are as high as in non-noise-exposed areas. However, this assumption is not realistic.

Begou and Kassomenos [55] compared DALY calculations made with a noise mapping of an urban area in Greece to noise levels determined according to WHO recommendations. Using the noise mapping, which included noise levels above a mapping threshold of 45 dB(A), 15% of the exposed population was highly annoyed, and 8% were highly sleep-disturbed. Using the WHO guidance document to determine health effects at noise >55 dB(A), “only” 9% of the exposed population was highly annoyed and 4% were highly sleep-disturbed. Schreckenberg, et al. [17] also considered road-traffic noise over 40 dB(A) in their burden of disease study of Düsseldorf, Germany; finding 11.82% of residents were highly annoyed and 2.30% were highly sleep-disturbed. Despite using a more conservative annoyance curve, our results for Hesse (including cities as well as rural areas) point in the same direction (8.04% highly annoyed and 2.29% highly sleep-disturbed).

Considering only the Hessian population included in the EU Environmental Noise Directive mapping of road noise resulted in a 76% decrease in DALYs due to cardiovascular diseases and depression. It also resulted in a nearly 80% decrease in DALYs due to annoyance, and a 70% decrease in DALYs due to sleep disturbance. This assumes there is no increased health risk below an L_DEN_ of 55 dB(A) or L_night_ of 45 dB(A) and the population not included in the EU Environmental Noise Directive mapping is not exposed to road noise. As both of these assumptions are unlikely, the EU noise estimates probably underestimate the actual burden of disease. This comparison highlights the advantages of the more comprehensive noise “PLUS-Mapping”.

Another assumption needed to calculate the burden of disease attributable to noise is the risk estimates that describe the dose-response relationship between noise and health outcomes. We chose to use the NORAH risk estimate of 1.024 per 10 dB(A) L_24h_ (95% CI 1.016–1.033) for our main estimation of cardiovascular disease risk attributable to noise. Van Kempen, et al. [3] estimated higher relative risks (RR) for incident ischemic heart diseases and incident stroke of 1.08 per 10 dB(A) L_DEN_ (95% CI 1.01–1.15) and 1.14 per 10 dB(A) L_DEN_ (95% CI 1.03–1.25), respectively, based on a meta-analysis of studies. Using the larger RR (and higher counterfactual of 53 dB[A]) in a sensitivity analysis resulted in more DALYs (7890 versus 5970). Using estimates for ischemic heart disease prevalence (RR per 10 dB(A) = 1.24, 95% CI 1.08–1.42) and mortality (RR per 10 dB(A) = 1.05, 95% CI 0.97–1.13) reported by van Kempen, et al. [3] to calculate YLL and YLD separately, would likely further change the estimated burden of disease due to ischemic heart disease. However, the risk estimates for prevalent stroke and stroke mortality reported by van Kempen, et al. [3] were not statistically significant.

One difference between our study and previous studies estimating the proportion of the population highly annoyed by road-traffic noise was our choice of the estimation model. We chose to use the more conservative dose-response relationship estimated by Guski et al. [6], which excluded studies from the Alps and Asia. As stated in Section 2.5, we based this decision on the fact that the annoyance curves have a counter-intuitive U-form at lower noise levels. According to the curve determined with all available studies (“WHO full dataset road”) around 9% of the population exposed to 40 dB L_DEN_ are highly annoyed, but the %HA begins to decrease as noise increases and reaches a nadir of 8% at around 45 dB(A). The %HA begins to increase again at L_DEN_ over 45 dB(A), so that the 9% HA is regained at about 53 dB(A) and increases to 11% at about 55 dB(A). In other words, the annoyance stays more-or-less constant in the 15 dB-range from 40 to 55 dB. Applied to the real-world situation, this suggests road-traffic noise annoyance in the range 40 to 55 dB(A) is rather high but quite insensitive to the actual noise level. The annoyance at higher levels increases moderately compared to the starting value at 40 dB(A).

### 4.2. Impact of Future Noise Reduction

Schreckenberg, et al. [17] simulated the impact of reducing urban speed limits to 30 km/h and using noise-reducing asphalt on health in Düsseldorf. The simulations showed that these interventions had little impact on the percentage of noise-exposed residents. Nevertheless, the interventions reduced DALY due to cardiovascular heart disease by 9.8% and reduced the DALY estimates that also included annoyance and sleep disturbance by 8.4%.

We considered more global and aggressive noise-abatement measures that could reduce road-traffic noise by about 3 dB(A). In addition to reducing urban speed limits to 30 km/h and using noise-reducing asphalt, this would include reducing automobile traffic by promoting the use of collective transport, bicycles, and walking and replacing cars with combustion motors with electric ones. The presented calculations found results comparable to previous calculations of Seidler, et al. [41] on the potential health impact of noise reduction scenarios concerning cardiovascular disease (−24% decrease in persons affected). According to our projections, such a noise reduction reduces the disease burden for (ischemic) cardiovascular diseases attributable to road-traffic noise by about 25%. In contrast, our previous estimates found reducing road-traffic noise L_DEN_ > 43.3 dB(A) by 3 dB would result in an 11% decrease in the number highly annoyed and a 13% decrease in the number of sleep-disturbed residents. Based on our current analyses, we find a 3-dB reduction of road-traffic noise would reduce DALYs per year due to annoyance and to sleep disturbances by 22% and 28%, respectively. This difference resulted mainly from divergent analysis methods. Our previous estimates set exposure levels below 40 dB(A) to 40 dB(A), a value where 9% were estimated to be highly annoyed. The current analysis considered 40 dB(A) to be a threshold for annoyance and sleep disturbances, and we attributed no annoyance or sleep disturbances to people exposed to lower noise levels.

Considering a hypothetical 3-dB reduction in noise was a rough approximation of a potentially achievable road-noise reduction. This is also no small difference, because a 3-dB(A) reduction in noise is equivalent to a halving of the sound energy. Transitioning to electrically powered cars may contribute to the noise reduction in urban areas. Verheijen and Jabben [21] predict moving to an entirely electric fleet of cars will reduce road-traffic noise by 3 to 4 dB(A) in urban areas. However, the effect of going electric would be negligible on intercity roads because tire noise dominates at speeds over 50 km/h. Goals set out by the Sustainable and Smart Mobility Strategy of the European Union for 2030 envisions a multimodal transportation system that emphasizes sustainable modes of transportation, such as collective transport, bicycling, and walking [56]. This will also help reduce noise levels outside of cities. Investing in infrastructure for bicyclists and pedestrians has the added benefit of directly improving cardiovascular health and reducing mortality [57].

### 4.3. Limitations

Due to a lack of prevalence and mortality data for individual German states, we used the WHO DALY estimates for Germany. This pragmatic choice had advantages. We benefitted from the correction for comorbidities and modeling of YLD for myocardial infarction with disability weights that decrease a few days after a non-fatal heart attack. We were unable to recreate these calculations with data available for Hesse. However, the German DALY estimate may not be accurate for Hesse. For example, the German DALY estimates for depressive disorders may underestimate the DALYs for Hesse. According to data based on the 2014/2015 GEDA study, the 12-month prevalence of depression was 9.3% in Hesse and 8.1% in Germany [58]. On the other hand, the 2015 rate of myocardial infarctions in Hesse (337.65 per 100,000) was similar to the German average (347.84 per 100,000) [59]. Thus, the cardiovascular disease DALYs for Germany should provide a reasonable estimate of the Hessian DALY.

While using risk estimates from a large study with routine data conducted in a population similar to the study population was advantageous for our calculations, the confidence of evidence is generally higher for the results of well-conducted systematic reviews. Therefore, we compared the results based on the dose-response relationship determined from the NORAH study on disease risks with the results from the WHO systematic review on cardiovascular disease [3,30]. The dose-response per 10 dB was higher for ischemic diseases and stroke, possibly due to the fact that some of the studies included in the WHO meta-analysis did not consider noise as low as L_DEN_ = 40 dB(A). The weighted average of lower noise levels from these reviews was L_DEN_ = 53 dB(A) [30]. This might have resulted in the steeper increase in risk per 10 dB(A) (8% versus 2.4% increase per 10 dB(A)). Using the WHO risk estimate results (with higher confidence of evidence) resulted in 1920 more DALYs due to cardiovascular disease. Therefore, our estimates may be conservative.

We decided to include depression in this analysis, despite of fact that the pooled effects from meta-analyses were not statistically significant [5,31]. Although the level of confidence of evidence for depression is very low [26,31], we chose to calculated DALYs for depression based on the fact that Dzhambov and Lercher [31] found a significant dose-response relationship at levels over 55 dB. Additionally, more recent prospective studies with a lower risk of bias report increased risk estimates (although these are not always statistically significant) [5]. Although systematic reviews have a higher level of evidence, we also chose to use the 4.1% risk increase per 10 dB(A) for depression from a single German study of routinely collected data [42]. However, pooled risk estimates from recent reviews describe a similar dose-response relationship of 3 and 4% increase per 10 dB(A), so this choice should have no substantial impact on this estimate.

With regard to depression, especially for females, the prevalence of depression is high in the age group below 40 years. For Germany, the “German Health Interview and Examination Survey for Adults in 2009–2012” estimated the 12-month prevalence of depression was 15.6 (11.3–21.0) for females aged 18–34 years, 11.0 (8.1–14.7) for females aged 35–49 years, and 5.0 (3.5–7.1) for females between 50–65 years in [60]. For males, the 12-month prevalence was around four-to-five and more evenly distributed in all age groups. Since we included risk estimates estimated for people ≥40 years and applied these to the DALYs in the similarly aged population, the calculated DALYs may underestimate the total burden of disease due to depression in the population, assuming the relationship between road-traffic noise and depression is confirmed in future studies for people of all ages. If we assume the risk of depression caused by road-traffic noise is the same for people of all ages, 1538 DALYs (95% CI 1168 to 1867) can be attributed to road-traffic noise for the entire population. However, we do not know if younger age groups react similarly to road noise. Future studies considering traffic-noise and depressive disorders should incorporate all age groups and differentiate between males and females.

Another potential limitation to our estimates is how we extracted WHO DALY estimates for the German population over the age of 40 years. The WHO DALYs report combines people aged 40 to 49 years with persons aged 30 to 39 years, we halved these estimates for our calculations. This assumption that DALYs were uniformly distribution among 30-to-49-year-olds will likely underestimate the DALYs due to cardiovascular disease [61].

For our calculations of DALYs attributable to a quieter road traffic scenario, we assumed that the total level of DALYs remained unchanged under the lower noise conditions, and only the proportion attributable to noise (PAF) changed. However, a noise reduction and the resulting reduction in noise-related disease would also lower the overall burden of disease in the population. We did not correct this lowered burden, so our estimates for the noise-reduction scenario may slightly overestimate the potential reduction of health burden.

Moreover, we used the most recent road noise data available for Germany, but changes in road-traffic patterns over time are possible. With regard to the COVID-19 pandemic, a decrease in road traffic leading to changes in air pollution has been observed by some studies [62]. For example, a decrease in road-traffic volume by 37% and road occupancy by 52% has been shown during the Stay Home Order (SHO) in Washington state [63]. Doucette, et al. [64] observed a 43% decrease in mean daily vehicle miles traveled in Connecticut during the SHO. Lower mobility during the pandemic has also been reported for European countries [65,66]. However, these changes may be temporary, and the overall effect on noise maps still has to be analyzed.

Finally, in the literature, the assumptions for the calculations of DALYs attributable to road-traffic noise often differ, making a direct comparison difficult. A more homogenous procedure for future studies is desirable.

## 5. Conclusions

A majority of the population of Hesse lives in areas where they are exposed to road-traffic noise levels over 40 dB(A). The health effects of road-traffic noise contribute to the burden of disease experienced due to cardiovascular disease and depressive disorders. Also, the widespread loss of well-being due to annoyance and sleep disturbances accounts for a substantial loss of healthy life years. Measures that reduce road-traffic noise by 3 dB(A) can reduce the burden of disease attributable to road-traffic noise by about one-fourth. Reducing road-traffic noise by lowering urban speed limits and using quieter asphalt will help reduce noise-related diseases. Further public health benefits will be gained by using sustainable modes of transportation, such as collective transport, bicycling, walking, and electric vehicles.

## Figures and Tables

**Table 1:** Relative risks for PAF calculations and the publication source.

Disease Group and Source of RR	ICD-10 Codes Used in the Publications	RR per 10 dB(A)	Noise Metrics
Incident Cardiovascular Diseases ^1^ [41]	I11, I13.0, I13.2, I21, I50, I61, I63, I64	1.024 (95% CI 1.016–1.033)	L_24h_ counterfactual: ≤40 dB(A)
Sensitivity Analysis (Sens1a): Incident Ischemic Heart Diseases ^2^ [3,30]	Not reported in the publication	1.08 (95% CI 1.01–1.15)	L_DEN_ counterfactual: ≤53 dB(A)
Sensitivity Analysis (Sens1b): Incident Stroke ^2^ [3,30]	Not reported in the publication	1.14 (95% CI 1.03–1.25)	L_DEN_ counterfactual: ≤53 dB(A)
Incident Depressive Disorders ^1^ [32]	F32, F33, F34.1, F41.2	1.041 (95% CI 1.031–1.050)	L_24h_ counterfactual: ≤40 dB(A)

^1^ Based on the NORAH study on disease risks (main analysis) conducted in Hesse; ^2^ based on WHO reviews.

**Table 2:** The DALYs due to cardiovascular diseases and depressive disorders attributable to all risks in Germany and Hesse (people age ≥ 40 years) used for the various analyses. The shaded rows were used for the calculation of DALYs attributable to road-traffic noise.

Population	Exposure	Outcome ^1^	Time	Burden of Disease (DALY) Source: WHO [46]
Main Analysis (and Sensitivity Analysis 3 and additional analysis)				
All Germans	none	CVD	2015	3,963,058
Germans aged ≥ 40 years	none	CVD	2015	3,859,243
Hessians aged ≥ 40 years	none	CVD	2015	287,321
All Germans	none	Depressive disorders	2015	586,898
Germans aged ≥ 40 years	none	Depressive disorders	2015	370,764
Hessians aged ≥ 40 years	none	Depressive disorders	2015	27,603
Sensitivity Analysis 1a				
All Germans	none	IHD	2015	2,577,937
Germans Aged ≥ 40 years	none	IHD	2015	2,513,610
Hessians Aged ≥ 40 years	none	IHD	2015	187,138
Sensitivity Analysis 1b				
All Germans	none	Stroke	2015	1,001,054
Germans Aged ≥ 40 years	none	Stroke	2015	964,478.5
Hessians Aged ≥ 40 years ^1^	none	Stroke	2015	71,805

^1^ NORAH study on health risks defined cardiovascular diseases (CVD) as ICD-10: I10–I15, I21, I60–I69, and WHO DALYs are for hypertensive heart disease and heart failure (ICD-10: I10–I15), ischemic heart disease (I20–I25), and stroke (I60–I69); IHD: Ischemic Heart Disease.

**Table 3:** Road-noise distribution in Hesse and the population-attributable fraction of cardiovascular diseases, ischemic heart disease, and depressive disorders for road-traffic noise.

L_DEN_	L_24h_^1^	N ^2^	%	Cardiovascular Diseases RR (Main Analysis) ^3^	Ischemic Heart Disease RR (Sens1a) ^4^	Stroke RR (Sens1b) ^5^	Depressive Disorders RR (Main Analysis) ^6^
≤43.3	≤40	1,262,833	20.8%	1.00	1.0	1.0	1.00
>43.3 to 48.3	>40 to 45	1,392,745	22.9%	1.01	1.0	1.0	1.01
>48.3 to 53.3	>45 to 50	1,224,661	20.2%	1.02	1.0	1.0	1.03
>53.3 to 58.3	>50 to 55	789,863	13.0%	1.03	1.02	1.04	1.05
>58.3 to 63.3	>55 to 60	537,212	8.8%	1.04	1.06	1.11	1.07
>63.3 to 68.3	>60 to 65	550,616	9.1%	1.05	1.10	1.18	1.09
>68.3 to 73.3	>65 to 70	229,583	3.8%	1.07	1.15	1.26	1.12
>73.3	>70	90,135	1.5%	1.08	1.19	1.35	1.14
				PAF = 2.1%	PAF = 2.5%	PAF = 4.4%	PAF = 3.5%

^1^ L_24h_ derived by subtracting 3.3 dB(A) from L_DEN_ [50]; ^2^ population included in the 2017 “PLUS-Mapping” of Hesse; ^3^ risks shown for the middle of the categories starting from L_24h_ values of 40 dB(A) using the dose-response relationship for cardiovascular diseases: 1.024 per 10dB (A) [41]. The analysis used finer 0.1-dB(A) categories for calculating PAF; ^4^ risks shown for the middle of the categories starting from L_DEN_ values of 53 dB(A) using the dose-response relationship for ischemic heart disease: 1.08 per 10 dB(A) [3,30]. The analysis used finer 0.1-dB(A) categories for calculating PAF; ^5^ risks shown for the middle of the categories starting from L_DEN_ values of 53 dB(A) using the dose-response relationship for stroke: 1.14 per 10 dB(A) [3,30]. The analysis used finer 0.1-dB(A) categories for calculating PAF; ^6^ risks shown for the middle of the categories starting from L_24h_ values of 40 dB(A) using the dose-response relationship for depressive disorders: 1.041 per 10 dB(A) [32]. The analysis used finer 0.1-dB(A) categories for calculating PAF.

**Table 4:** The DALYs due to cardiovascular diseases and depressive disorders attributable to road-traffic noise in Germany and Hesse (people age ≥ 40 years).

	PAF (95% CI)	DALYs in Hesse (95% CI)	DALY per 100,000
Cardiovascular Diseases [41] ^1^	2.1% (1.4% to 2.8%)	5970 (3996 to 8171)	173.0 (115.8 to 236.8)
Sensitivity Analysis 1a: Ischemic Heart Disease [3,30]	2.5% (0.4% to 4.7%)	4739 (599 to 8784)	137.4 (17.4 to 254.6)
Sensitivity Analysis 1b: Stroke [3,30]	4.4% (1.0% to 7.7%)	3151 (688 to 5526)	91.3 (19.9 to 160.2)
Depressive Disorders [32]	3.5% (2.7% to 4.3%)	971 (738 to 1179)	28.2 (21.4 to 34.2)

^1^ WHO DALYs for hypertensive heart disease and heart failure (ICD–10: I10–I15), ischemic heart disease (I20–I25), and stroke (I60–I69).

**Table 5:** Estimated number of residents highly annoyed due to road-traffic noise and respective DALYs in Hesse (all ages).

	Highly Annoyed in Hesse	DALYs in Hesse	DALY per 100,000
L_DEN_ ≥ 40 dB(A)	490,000 (8.04%)	9800	160.8
Sensitivity Analysis 2: Formula (9) included Alpine and Asian studies	684,108 (11.23%)	13,682	224.5

**Table 6:** Estimated number of residents highly annoyed due to road-traffic noise and respective DALYs in Hesse (all ages).

	Highly Sleep Disturbed in Hesse	DALYs in Hesse	DALY per 100,000
L_night_ ≥ 40 dB(A) [4]	139,427 (2.29%)	9760	160.2

**Table 7:** DALY calculations based on EU Environmental Noise Directive mapping for the population in Hesse over 40 years of age.

L_DEN_	L_24h_^1^	N	%	Cardiovascular Diseases RR ^2^	Depressive Disorders RR ^3^
≤55	≤51.7	5,474,104	89.5%	1.00	1.00
55 to ≤60	51.7 to ≤56.7	280,251	4.6%	1.03	1.06
60 to ≤65	56.7 to ≤61.7	165,586	2.7%	1.05	1.08
65 to ≤70	61.7 to ≤66.7	123,528	2.0%	1.06	1.10
70 to ≤75	66.7 to ≤71.7	63,997	1.0%	1.07	1.12
>75	>71.7	8737	0.1%	1.08	1.15
				PAF = 0.5%; DALY = 1400	PAF = 0.8%; DALY = 231

^1^ L_24h_ derived by subtracting 3.3 dB(A) from L_DEN_ [50]; ^2^ risks calculated for the median of the categories starting from L_24h_ values of 40 dB(A) using the dose-response relationship for cardiovascular diseases: 1.024 per 10 dB (A) [41]; ^3^ risks calculated for the median of the categories starting from L_24h_ values of 40 dB(A) using the dose-response relationship for depressive disorders: 1.041 per 10 dB(A) [32].

**Table 8:** The DALYs calculations based on EU Environmental Noise Directive mapping.

L_DEN_	N	Highly Annoyed (HA)			L_night_	N	Highly Sleep Disturbed (HSD)		
		%HA	*n*	DALY			%HSD	*n*	DALY
<55	5,474,104	0	0	0	<45	5,331,342	0	0	0
55 to <60	280,251	8.5%	23,918	478.4	45 to <50	372,112	3.5%	13,076	915.3
60 to <65	165,586	14.7%	24,314	486.3	50 to <55	207,676	5.1%	10,687	748.1
65 to <70	123,528	23.3%	28,804	576.1	55 to <60	134,101	7.4%	9934	695.4
70 to <75	63,997	34.4%	22,038	440.8	60 to <65	61,708	10.3%	6356	444.9
≥75	8737	48.0%	4197	83.9	65 to <70	9264	13.8%	1280	89.6
					≥70	487	18%	88	6.1
Total				2065					2899

**Table 9:** The total DALYs attributable to past/current road-traffic noise patterns and assuming a scenario with 3-dB(A) noise reduction in the time before the reference year.

	Past/Current Conditions		Scenario with 3-dB Reduction		DALY Change for Hesse
	Number of Persons with Disease in Hesse (%)	DALYs in Hesse	Number of Persons with Disease in Hesse (%)	DALYs in Hesse 3-dB Reduction Scenario	
Cardiovascular Diseases ^1^ (≥ 40 years of age)		5970		4508	−1462 (−24%)
Depressive Disorders (≥40 years of age)		971		735	−236 (−24%)
Highly Annoyed	490,000 (8.04%)	9800	380,499 (6.24%)	7610	−2190 (−22%)
Highly Sleep Disturbed	139,427 (2.29%)	9760	100,439 (1.65%)	7031	−2729 (−28%)
Total		26,501		19,884	−6617 (−25%)
DALY per 100,000 ^2^		434.9		326.3	

^1^ Hypertensive heart disease and heart failure (ICD-10: I10–I15), ischemic heart disease (I20–I25), and stroke (I60–I69); ^2^ calculated using population of Hesse in 2015 = 6,093,888 [51].

## Data Availability

Our analyses are, in part, based on published data [3,30,32,41,46,47]. Data on the number of residents exposed to road traffic noise based on the “PLUS–Mapping” are available for valid research purposes on request from HLNUG. The data are not publicly available due to copyright restrictions.
